# miRNA-Mediated Regulation of *Meloidogyne arenaria* Responses in Wild *Arachis*

**DOI:** 10.3390/ijms262210824

**Published:** 2025-11-07

**Authors:** Patricia Messenberg Guimaraes, Andressa da Cunha Quintana Martins, Roberto Coiti Togawa, Mario Alfredo de Passos Saraiva, Ana Luiza Machado Lacerda, Ana Cristina Miranda Brasileiro, Priscila Grynberg

**Affiliations:** 1 EMBRAPA Recursos Genéticos e Biotecnologia, Brasília 70770-917, Brazil; andressacqm@gmail.com (A.d.C.Q.M.); roberto.togawa@embrapa.br (R.C.T.); mario.saraiva@embrapa.br (M.A.d.P.S.); analuiza.lacerda@gmail.com (A.L.M.L.); ana.brasileiro@embrapa.br (A.C.M.B.); priscila.grynberg@embrapa.br (P.G.); 2 Instituto Nacional de Ciência e Tecnologia—INCT PlantStress Biotech-Embrapa, Brasília 70770-917, Brazil

**Keywords:** root-knot nematodes, *Meloidogyne* spp., NLR genes, small RNA, *Arachis stenosperma*

## Abstract

MicroRNAs (miRNAs) are key post-transcriptional regulators of plant development and stress responses, with many being conserved across diverse plant lineages. In this study, we investigated the expression profiles of miRNAs and their corresponding target genes in *Arachis stenosperma*, a wild peanut relative that exhibits robust resistance to root-knot nematodes (RKN). Small RNA sequencing of nematode-infected roots identified 107 miRNA loci, of which 93 corresponded to conserved miRNA families and 14 represented novel candidates, designated as miRNOVO. Among these, 18 miRNAs belonging to 11 conserved families were identified as differentially expressed (DEMs). Notably, *miR399* and *miR319* showed the highest upregulation (logFC = 4.25 and 4.20), while *miR393* and *miR477* were the most downregulated (logFC = −0.83 and −0.79). Integrated analysis of miRNA and transcriptome data revealed several regulatory interactions involving key defense-related genes. These included NLR genes targeted by *miR393* and *miR477*, hormone signaling components such as the auxin response factor *ARF8* targeted by *miR167*, and the growth regulator *GRF2* targeted by *miR396*. Additionally, *miR408* was predicted to target *laccase3*, a gene involved in the oxidation of phenolic compounds, lignin biosynthesis, copper homeostasis and defense responses. Remarkably, four immune receptor genes belonging to the nucleotide-binding site leucine-rich repeat (NLR) family displayed inverse expression patterns relative to their regulatory miRNAs, suggesting miRNA-mediated post-transcriptional control during the early stages of nematode infection. These findings reveal both conserved and species-specific miRNA–mRNA modules associated with nematode resistance in *A. stenosperma*, highlighting promising targets for developing RKN-tolerant peanut cultivars through miRNA-based strategies.

## 1. Introduction

MicroRNAs (miRNAs) participate in various plant developmental processes, gene expression regulations, and complex cellular mechanisms such as defense strategies, intra- and extracellular stress responses and growth initiation [1]. They are fundamental players in broad-spectrum diseases, as pathogen attack triggers massive miRNA changes in the host causing alteration or silencing of diverse phytohormone pathways, thus regulating plant immunity [2]. miRNAs are single-strand small non-coding RNAs, typically 21–24 nucleotides in length, that originate from miRNA genes and function at the post-transcription level by base pairing with cognate mRNA, degrading or inhibiting mRNA translation [3]. They are evolutionarily highly conserved in plant species, with each species harboring primarily conserved miRNAs, mostly involved in regulating common plant developmental processes, and some species-specific miRNAs, regulating special trait development, such as nodule development and symbiotic nitrogen fixation in legumes [4,5]. The expression of miRNAs can vary depending on cell type, tissue, genotype, or environmental condition, thereby regulating the expression of their target genes. For instance, miRNAs modulate biotic and abiotic stress-responsive genes, including pathogenesis-related (PR) and resistance (R) genes, and those involved in drought, aluminum and salinity tolerance mechanisms [6,7,8,9].

Peanut (*Arachis hypogaea*) is one of the most important legume crops in the world, grown widely for both oil and protein production (http://www.fao.org/faostat/, accessed on 29 October 2025). Their seeds also offer health benefits, being rich in heart-healthy oleic and linoleic acid, resveratrol, fiber and folic acid, and easily digested protein [9]. Nevertheless, destructive diseases including early and late leaf spot, spotted wilt disease, white mold, and nematode infection affect various plant parts and can drastically reduce peanut yield and quality [10,11]. In contrast, peanut wild relatives (*Arachis* spp.) have evolved and adapted to a wide range of environments, and harbor high levels of resistance to many pathogens, including the highly damaging root-knot nematode (RKN) *Meloidogyne arenaria*, and tolerance to drought [12,13,14,15]. Thus, wild *Arachis* species constitute an important genetic reservoir for resistance and tolerance traits that can be seized and transferred to cultivated peanut or other legume crops by marker-assisted breeding or transgenic approaches [16,17].

Several studies have demonstrated the involvement of peanut miRNAs in the regulation of key developmental processes, including gynophore formation [18], embryo abortion [19], embryogenesis and pod development [20], anthocyanin biosynthesis [21], and seed expansion [22]. Additionally, miRNAs have been implicated in peanut responses to a wide range of biotic stresses, such as bacterial wilt (*Ralstonia solanacearum*) [23], *Aspergillus flavus* infection [24,25], *Sclerotium rolfsii* [26] and RKNs [27], as well as abiotic challenges, including nitrogen and potassium deficiencies [28], drought and heat stress [29], cold [30] and aluminum toxicity [9]. In contrast, relatively few studies have investigated miRNAs and their target genes in wild *Arachis* species. Notably, Zhao et al. [23] reported contrasting miRNA expression profiles in response to bacterial wilt between the wild species *A. glabrata*, which shows resistance to soil-borne pathogens, and the susceptible cultivated peanut, and Garg et al. [31] conducted a comprehensive genome-wide analysis of key components involved in miRNA biogenesis and target gene regulation in *A. duranensis* and *A. ipaënsis*, the wild progenitors of peanut. Both works provided valuable insights into the small RNA-mediated regulatory networks in these species.

In this study, we analyzed the miRNA transcriptome of *A. stenosperma*, a wild species highly resistant to *M. arenaria*, to identify pathogen-responsive miRNAs and predict their corresponding target genes. Our results reveal complex miRNA-mediated regulatory networks underlying RKN resistance in this wild species and offer valuable insights into the molecular mechanisms of its robust defense against nematode infection. As growing evidence shows that pathogen-responsive small RNAs fine-tune plant immunity by silencing negative regulators or activating positive ones, our findings highlight promising candidates for the application of small RNA–based technologies, such as artificial microRNAs (amirRNAs), to develop peanut cultivars with durable resistance to *M. arenaria* and other nematodes.

## 2. Results

### 2.1. miRNA Sequencing and Analysis

Overall, six miRNA libraries were constructed, consisting of three libraries from *A. stenosperma* roots inoculated with *M. arenaria* (AsINOC) collected at 6 days after inoculation (DAI) and three of non-inoculated control (AsCTR), with an average number of raw reads of 30 and 32.9 Mb, respectively (Supplementary Table S1). Since a fully annotated genome of *A. stenosperma* is not yet available, after contaminants removal and size selection (20–24 nt), transcript reads were mapped into *A. duranensis* reference genome (https://peanutbase.org/, accessed on 29 October 2025; [32]). In average, 2.6% of *A. stenosperma* miRNA reads, which represents 53.8% of 20–24 nt sequences, mapped uniquely to the heterologous *A. duranensis* genome (Supplementary Table S1).

### 2.2. A. stenosperma miRNAs Expression During M. arenaria Infection

**Figure 1:**
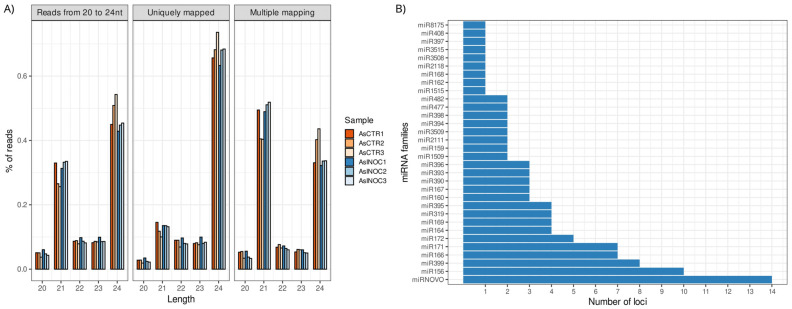
Overview of sRNA-Seq data in *Arachis stenosperma* roots infected with *Meloidogyne arenaria*: (**A**) Percentage of reads mapped to the *A. duranensis* reference genome from *A. stenosperma* infected (AsINOC—shades of blue) and control (AsCTR—shades of red) libraries: (i) total mapped reads between 20 and 24 nt; (ii) uniquely mapped reads; and (iii) ambiguously mapped reads (**B**) Number of miRNA loci per miRNA family identified in *A. stenosperma*.

The size distribution of miRNA-mapped reads from *A. stenosperma* revealed prominent peaks at 21 and 24 nucleotides, consistent with canonical plant miRNA profiles. Across all libraries, 24-nt miRNA reads represented the highest proportion of both total and uniquely mapped reads to the reference genome, whereas 21-nt reads were more frequently associated with multi-mapping loci, in line with expectations for plant-derived small RNA datasets (Figure 1A). A subtle trend was observed toward a higher number of 21-nt reads in inoculated samples and 24-nt reads in control samples; however, this difference was not statistically significant. These results are consistent with those reported for other plant species, including *Arabidopsis thaliana*, *Medicago truncatula*, *Oryza sativa*, *Populus* spp. and *Citrus trifoliate*, where sequences with 24 nt in length dominated the whole miRNA transcriptome [33].

The prediction of miRNA precursors using the *A. duranensis* reference genome identified 107 distinct miRNA loci, of which 93 belonged to conserved miRNA families and 14 represented novel candidates, designated as miRNOVO (Figure 1B; Supplementary Table S2). These miRNOVO sequences were classified as novel because they exhibited no detectable homology in the miRBase database (release 22.1; e-value < 10^−4^) [34], yet fulfilled the established structural and biogenetic criteria for authentic miRNAs as outlined by Axtell and Meyers [35].

To ensure that the miRNAs identified in the *A. stenosperma* libraries, including miRNOVO, were of plant origin and not derived from nematode sequences, we compared all plant miRNA sequences against *M. arenaria* miRNA transcripts (unpublished data). No identical matches were detected, confirming that the *A. stenosperma* miRNAs did not result from nematode contamination.

Predicted miRNAs in *A. stenosperma* were distributed across 31 conserved families, of which 25 are broadly conserved in plants, one is specific to Fabaceae (miR1509), and three are exclusive to the genus *Arachis* (miR3508, miR3509, and miR3515). Among these, miR156, miR399, miR166, and miR171 showed the highest genomic representation, with 10, 8, 7, and 7 loci, respectively (Figure 1B).

### 2.3. A. stenosperma Differentially Expressed miRNAs (DEMs) in Response to M. arenaria Infection

**Figure 2:**
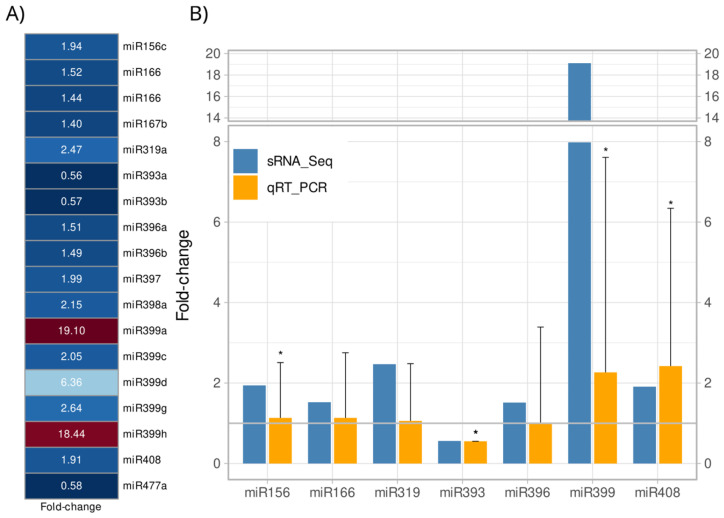
Differentially expressed miRNAs (DEMs) in *Arachis stenosperma* roots in response to *Meloidogyne arenaria* infection. (**A**) Heatmap showing the relative expression of 18 DEMs (FDR < 0.05) in *A. stenosperma* based on their in silico average fold change (FC) values between *M. arenaria* infected and control roots. The red-blue scale indicates upregulation (red) and downregulation (blue); (**B**) Expression profiles of seven DEMs validated by qRT-PCR (Relative Quantification, RQ) (orange) compared to RNA-Seq-based fold change (FC) values (blue). Relative Quantification (RQ) values were calculated from the mean of three biological replicates (*n* = 9 total) and analyzed by Tukey’s test (* *p* ≤ 0.05).

A total of 18 miRNAs belonging to 11 miRNA conserved families were identified as differentially expressed miRNAs (DEMs) in *A. stenosperma* roots upon *M. arenaria* infection compared to non-inoculated control (FDR < 0.05) (Figure 2A). The majority of these DEMs (15 out of 18) were upregulated in response to nematode infection, while three miRNAs belonging to two families (miR393 and miR477) were downregulated (Figure 2A). Four miR399 loci and miR319 exhibited the highest levels of upregulation (logFC = 4.25, 4.20, 2.66 and 1.40 for miR399 and 1.30 for miR319)), whereas miR393 and miR477 showed the strongest downregulation (logFC = −0.83 and −0.80 for miR393 and −0.79 for miR477)) (Figure 2A; Supplementary Table S3). miR166 was by far the most abundant DEM, with up to 5.7 million reads in infected and control roots, followed by miR396 and miR159 (Supplementary Table S2).

To validate the in silico expression profiles of *A. stenosperma* DEMs, we analyzed the expression of seven miRNAs by quantitative reverse transcription-polymerase chain reaction (qRT-PCR) using the same miRNA samples extracted for sequencing, and specific primers designed following the stem-loop methodology described by Varkonyi-Gasic et al. [36] (Supplementary Table S4). Overall, the expression trends for the miRNAs in the qRT-PCR analysis supported the accuracy and reliability of the in silico analysis (Figure 2B). Notably, miRNAs previously associated with biotic stress responses, such as miR166, miR399 and miR408 were found to be upregulated in both analyzes. These miRNAs are known to regulate various stress-related pathways in plants, as miR166 is involved in stress signaling and vascular development [37], miR399 plays a central role in phosphate homeostasis and has been linked to defense responses [38], while miR408 is implicated in oxidative stress regulation and copper homeostasis [39]. The validation of these expression patterns through qRT-PCR underscores the potential roles of these miRNAs in mediating *A. stenosperma* responses to stress, particularly in the context of biotic challenges.

### 2.4. Correlation of Expression Profiles Between miRNAs and Their Target Genes

We identified 92 miRNA loci with at least one predicted target gene in the *A. duranensis* reference genome (Supplementary Table S5). These miRNAs belong to 29 conserved families and 14 novel sequences (miRNOVO), collectively targeting 584 loci corresponding to 435 different gene products in *A. duranensis*. While some miRNA families had numerous predicted target genes in *A. duranensis*, such as miR156 (78), miR172 (45) and miR396 and miR399 (25 each), miR2118 had only one target.

**Figure 3:**
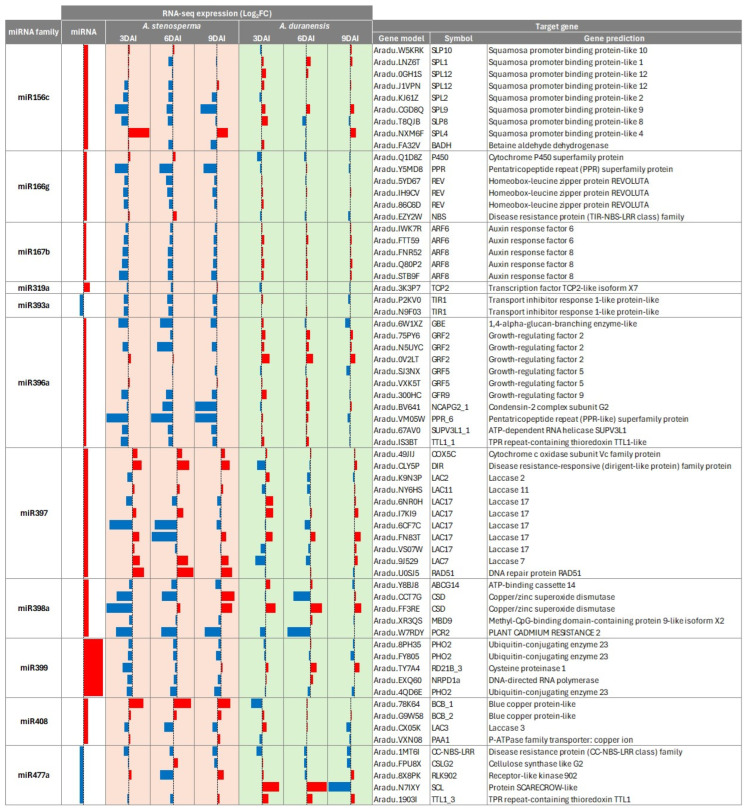
Heatmap of the RNA-Seq expression (Log_2_ FC) of *A. stenosperma* miRNAs and their target genes in the RKN resistant *A. stenosperma* and the more susceptible *A. duranensis*. Colum: (1) miRNA identity; (2) miRNA expression (Log_2_ FC); (3–8) RNA-Seq expression (Log_2_ FC) of target genes at three time-points (DAI) after *M. arenaria* inoculation in *A. stenosperma* (3, 6 and 9 DAI); (8–10) RNA-Seq expression (Log_2_ FC) of target genes at three time-points after *M. arenaria* inoculation in *A. duranensis* (3, 6 and 9 DAI); (9) miRNA target gene identity (ID) in *A. duranensis*; (10) symbol; (11) target gene annotation (https://www.peanutbase.org/, accessed on 29 October 2025).

To further explore the regulatory dynamics of *A. stenosperma* miRNAs, we compared the expression patterns of the previously identified DEMs and their predicted targets between the resistant *A. stenosperma* and the susceptible *A. duranensis*, using our RNA-Seq datasets across three post-inoculation time points (3, 6, and 9 DAI) [12,40]. Overall, 11 DEMs predicted a total of 65 target genes in the transcriptomes of both *A. stenosperma* and *A. duranensis* (Figure 3). Most miRNAs (nine) were upregulated in *A. stenosperma* during nematode infection, including members of the miR399 family, which showed significant expression changes in five isoforms (log2FC ranging from 1.03 to 4.25). In contrast, the most strongly downregulated miRNAs were miR393 and miR477, with log2FC values of −0.83 and −0.79, respectively (Figure 3).

Notably, several DEMs, particularly those associated with stress response pathways, exhibited contrasting target gene expression profiles between the RKN-resistant *A. stenosperma* and the more susceptible *A. duranensis*. These targets include key transcription factors and stress-related proteins such as SQUAMOSA Promoter Binding Protein-Like (SPL) genes, canonical targets of miR156; the homeobox-leucine zipper protein REVOLUTA, a well-characterized target of miR166; auxin response factors (ARFs), primarily regulated by miR167; transport inhibitor response 1, target by miR393 and growth-regulating factors (GRFs), targeted by miR396. Additionally, copper/zinc superoxide dismutase (Cu/Zn-SOD), a critical enzyme in reactive oxygen species (ROS) detoxification, is regulated by miR398. Predicted target genes encoding disease resistance proteins, such as dirigent-like protein (DIR) targeted by miR397, nucleotide-binding site leucine-rich repeat (NLRs) proteins regulated by miR166, and genes involved in oxidative stress responses, such as aldehyde dehydrogenase (ALDH) and cytochrome C oxidase (COX), which are targets of miR156 and miR397, respectively, also exhibited species-specific opposite expression patterns (Figure 3).

Other miRNA-target pairs, despite not exhibiting huge differences in their expression levels are also worth attention, such as those displayed by some of the laccase genes (LAC), known targets of miR397, and by PHO genes, known targets of miR399. miR399 was the most differentially expressed miRNA, with five isoforms showing induced expression in response to infection, while its target gene PHO2 displayed a mild but consistent downregulation in the resistant *A. stenosperma*. This comparative expression analysis highlighted several miRNA–target regulatory pairs that may underlie the distinct resistance responses observed between the two species, offering insights into the molecular mechanisms of nematode resistance in wild *Arachis*.

Representatives of six DEM families in *A. stenosperma* (miR156, miR166, miR319, miR396, miR399 and miR408) and their respective target genes had their expression behavior in silico validated by qRT-PCR analysis. For that, we used total RNA from *A. stenosperma* roots infected with *M. arenaria* at 6 DAI, which is the inflection time point of most RKN-responsive genes [12], and specific primers for each miRNA according to Varkonyi-Gasic et al. [36] (Supplementary Table S4). In addition to belonging to different conserved families, these DEMs were selected as their predicted target genes are well-known to be involved in response to stress, regulating different metabolic and regulatory pathways [41]. For instance, miR156, which target is a squamosa promoter binding protein-like 2 (SPL2), is involved in stress signaling and plant developmental process [42,43], miR166 targeting pentatricopeptide repeat (PPR)-like superfamily protein, is involved in biotic and abiotic stress [44], miR319, targeting transcription factor TCP2-like, is involved in leaf development and biotic stress responses [45,46], miR396, targeting RNA-binding protein (RBP), is implicated in miRNA binding and biogenesis [47], miR399 targeting ubiquitin-conjugating enzyme (PHO2), has a role in phosphate homeostasis and virus defense response [48,49] and miR408 targeting laccase genes (LAC3), which are enzymes involved in oxidation of phenolic compounds, lignin biosynthesis, copper homeostasis, and defense responses [50].

**Figure 4:**
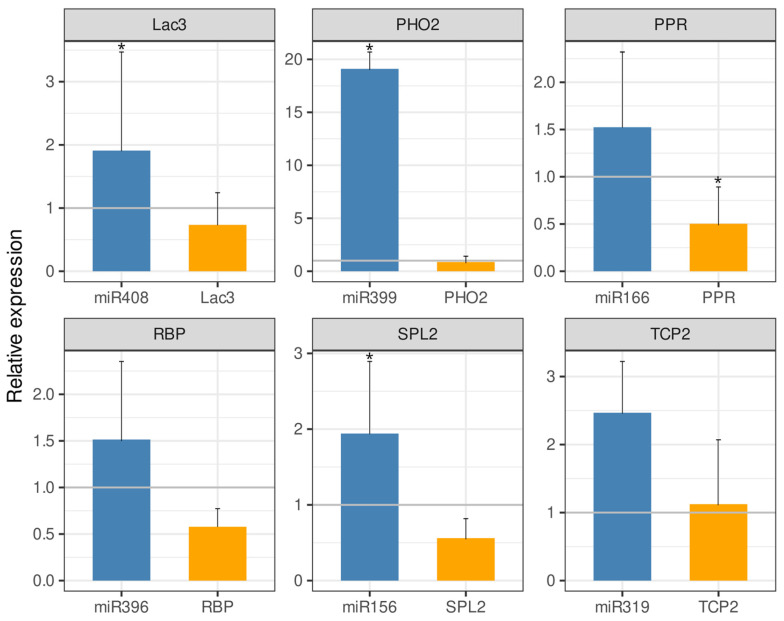
Expression of six *A. stenosperma* miRNAs and their respective target genes during *M. arenaria* infection by qRT-PCR. The relative quantification (RQ) of mRNA levels of small RNAs (miRNAs) and target genes in inoculated plants was normalized with non-stressed control samples using two miRNA reference genes for the miRNAs (U3 and U6) and two *Arachis* reference genes (60S and GAPDH), with RQ values above or below 1.0 indicating, respectively, up- or downregulated genes (* *p* ≤ 0.05).

All the six miRNAs selected were upregulated in response to *M. arenaria* infection, with a contrasting behavior compared to their respective target genes in *A. stenosperma* that produced lower transcript levels, confirming their in silico expression profiling. The exception was miR319 and its target gene, the transcription factor (TCP2), which were both slight upregulated at similar levels (Figure 4).

**Figure 5:**
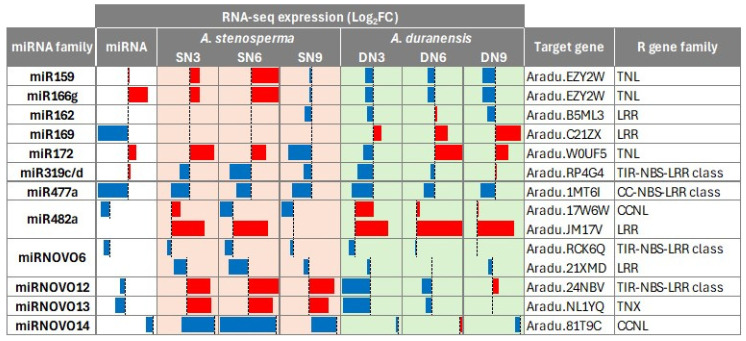
Heatmap of the RNA-Seq expression (Log_2_FC) of *A. stenosperma* miRNAs and their target NLR genes in *A. stenosperma* (SN) and *A. duranensis* (DN). Column: (1) miRNA identity; (2) miRNA expression (Log_2_FC); (3–5) RNA-Seq expression (Log_2_FC) of *A. stenosperma* target genes at three time-points after *M. arenaria* inoculation (3, 6, 9 DAI); (6–8) RNA-Seq expression (Log_2_FC) of *A. duranensis* target genes at three time-points after *M. arenaria* inoculation (3, 6 and 9 DAI); (9) target gene annotation (https://www.peanutbase.org/, accessed on 29 October 2025); (10) target NLR gene family. Values are Log2FC between inoculated and control samples (*p* < 0.05).

Markedly, 12 miRNAs identified in *A. stenosperma* during *M. arenaria* infection, including the differentially expressed miR166g and miR477a, were predicted to target resistance (R) genes from the nucleotide-binding site leucine-rich repeat (NLR) family (Figure 5). Interestingly, three of these miRNAs (miR482a, miRNOVO12, miRNOVO13) showed downregulation, while four of their corresponding target NLRs were upregulated in the resistant *A. stenosperma* in at least one of the early time points (3 and 6 DAI) of the RKN infection (Figure 5).

This contrasting expression behavior suggests a possible post-transcriptional regulation mechanism contributing to nematode resistance. Interestingly, the gene model Aradu.NL1YQ has previously been identified as a differentially expressed gene (DEG) in response to both *M. arenaria* infection and UV stress in *A. stenosperma*, while Aradu.EZY2W was responsive to UV stress [40,51].

These findings suggest that the downregulation of specific miRNAs at the early stages of nematode infection might contribute to the accumulation of their corresponding target NLR transcripts, thereby strengthening the plant’s immune response. This inverse expression pattern highlights a potential regulatory mechanism that contributes to *A. stenosperma* more robust defense against *M. arenaria* and possibly other root-knot nematodes.

**Figure 6:**
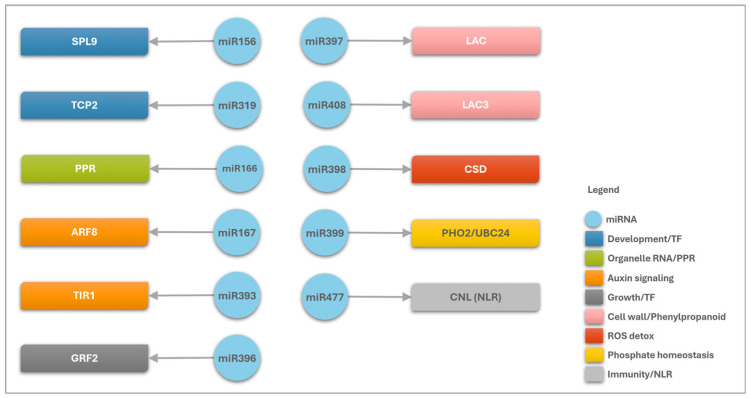
Schematic representation of *A. stenosperma* differentially expressed miRNA (DEM)–target interactions involved in the plant’s response to *M. arenaria* infection. Blue circles represent miRNAs, while colored squares indicate the functional categories of their target genes. Black arrows illustrate the regulatory relationships between miRNAs and their targets.

In general, the correlation of the DEMs and their predicted targets in *A. stenosperma* revealed their involvement in diverse stress-responsive pathways during *M. arenaria* infection (Figure 6). These miRNAs appear to regulate genes that are critical components of plant defense, including pathogen recognition via NLR resistance proteins, modulation of hormone signaling pathways such as jasmonic acid and salicylic acid, reinforcement of cell walls through dirigent-like proteins and laccases, mitigation of oxidative stress via oxidoreductases and superoxide dismutases, and maintenance of nutrient homeostasis through regulators such as PHO2 and PPR proteins (Figure 6). The involvement of these miRNA/target modules in diverse biological processes during *M. arenaria* infection in wild *Arachis* shows their relevant role in coordinating plant defense responses, highlighting their potential as valuable molecular markers or targets for the development of nematode-resistant cultivars through precision breeding or genetic engineering strategies.

## 3. Discussion

High-throughput sequencing and bioinformatics have revealed a diverse set of miRNAs and their targets in peanut, regulating key processes such as growth, development, and stress responses [22,29,52]. Recent studies further demonstrate their roles in modulating disease resistance pathways [24,25,27]. Currently, 11 conserved miRNAs from eight families and 12 peanut-specific sequences are listed in miRBase (http://www.mirbase.org/, accessed on 29 October 2025), underscoring the importance of miRNA-mediated regulation. However, despite the potential of wild *Arachis* species as sources of stress-resilient traits, their miRNA repertoires remain poorly explored. Although miRNA biogenesis proteins show strong evolutionary conservation, stress-specific regulatory differences suggest the existence of species- or stress-specific mechanisms [31]. Thus, investigating conserved and novel miRNAs and their targets in wild *Arachis* offers a promising approach to uncover regulatory elements that could enhance stress tolerance in cultivated peanut.

Disruption of specific miRNA biogenesis or function has been linked to increased resistance to both RKN and cyst nematodes (CN) nematodes, underscoring their importance in modulating plant susceptibility to phytonematodes [52,53,54]. Notably, a general downregulation of host miRNAs has been observed in galls induced by *Meloidogyne* spp. [55] and in CN-induced syncytia [56], with many of these miRNAs associated with stress responses [57]. This widespread repression of plant miRNAs in RKN-induced galls may reflect their role in regulating developmental processes that balance cell proliferation and differentiation within the complex gall structure. These massive miRNA changes triggered by pathogen attack has also been reported in other plant–pathogen interactions [2].

In this study, we identified 18 DEMs, belonging to 11 miRNA families, in the RKN-resistant *A. stenosperma* of which 15 were upregulated during *M. arenaria* infection. These included upregulated miRNAs associated with the regulation of cell growth and development, nutrient homeostasis, oxidative stress, and defense-related protein biosynthesis. Seven of these DEM families, miR156, miR167, miR393, miR396, miR398, miR399 and miR408, have previously been implicated in plant responses to RKN infection. miR156 has been reported to regulate Squamosa Promoter Binding Protein-like (SPL) genes in susceptible tomato (*Solanum lycopersicum*) roots infected with *M. incognita* [58]. In our study, we observed a negative correlation between miR156 and SPL transcript levels across four loci in *A. stenosperma*. The miR156/SPL regulatory module plays a central role in modulating immune-related genes, including Jasmonate-ZIM-domain (JAZ) proteins, which act as negative regulators of jasmonate signaling—a key pathway in plant defense [59]. miR156 upregulation leads to lower SPL levels, which in turn weakens SPL-JAZ repression and allows stronger jasmonic acid (JA) responses. Notably, *A. stenosperma* showed miR156 upregulation together with downregulation of four SPL loci, a pattern absent in susceptible *A. duranensis*. These data support a model in which RKN-induced miR156 represses SPLs, alleviates JAZ-based inhibition, and thereby enhances JA-mediated defense in the resistant species.

A particularly noteworthy finding is the upregulation of miR167, a microRNA known to target the auxin response factors ARF6 and ARF8. In this study, gene expression analysis reveals that these targets are downregulated in *A. stenosperma*, a resistant genotype, while showing an elevated expression in *A. duranensis*, which is susceptible to RKN infection. Previous studies in *A. thaliana* have established the role of auxin in gall development and nematode feeding site formation [60,61]. More recently, Noureddine et al. [62] demonstrated that miR167 and its targets ARF8A and ARF8B are critical regulators of gall formation in tomato. Their study identified the ARF8/miR167 regulatory module as being actively involved in the plant’s response to nematode infection, with upregulation of ARF8A and ARF8B in giant cells and surrounding gall tissues. This suggests that miR167-mediated repression of ARFs in resistant genotypes may serve as a defense strategy to suppress auxin signaling, thereby inhibiting the formation of nematode-induced feeding structures.

In addition to ARFs, the auxin receptor TIR1 (Transport Inhibitor Response 1), typically targeted by miR393, is also downregulated in the resistant *A. stenosperma* but not in the susceptible *A. duranensis*. When auxin binds TIR1 and other auxin F-BOX proteins, it promotes degradation of Aux/IAA repressors, releasing ARFs to activate auxin-responsive genes. This signaling pathway regulates root development, organogenesis, tropic growth, and stress adaptation in plants [63]. Here, we observed concurrent repression of both miR393 and its target gene *TIR*, likely reflecting restricted auxin signaling in response to nematode infection, a pattern not detected in susceptible species. This apparent decoupling may reflect a fine-tuned regulatory balance, wherein the plant modulates auxin perception independently of miR393 to avoid excessive suppression of auxin signaling. Such regulation could be essential for maintaining normal root development while still restricting nematode-induced cellular reprogramming. In resistant species, this strategy likely contributes to limiting gall formation and nematode establishment, without compromising overall root functionality.

Notably, members of the miR396 family, previously implicated in regulating parasitism by CN and RKN via the GRF (Growth Regulating Factor) pathway [53,64,65,66], were slightly -upregulated in *A. stenosperma* roots. In contrast, their GRF2 target genes were downregulated in this resistant genotype but upregulated in the more susceptible *A. duranensis*, suggesting miR396-mediated repression of growth-promoting genes may contribute to enhanced resistance. In a study involving *A. thaliana* infected with the cyst nematode *Heterodera schachtii*, elevated levels of miR396—resulting in the repression of GRF1 and GRF3—were associated with a reduction in syncytium size and the induction of nematode resistance [53]. These findings are consistent with the results obtained in the present study for the *A. stenosperma*-*M. arenaria* interaction.

Likewise, miR399, the most strongly upregulated miRNA in *M. arenaria*-infected *A. stenosperma* roots, exhibited inverse expression with its target gene, PHO2, a ubiquitin-conjugating enzyme involved in phosphate (Pi) homeostasis and protein turnover [67]. The PHO2 downregulation could facilitate increased phosphate mobilization, potentially support the metabolic demands of defense responses or alter nutrient availability in a way that is unfavorable to nematode development. Although miR399 has not been widely reported in RKN-related studies, previous reports have shown differential miR399 expression in tomato during *M. incognita* infection [58], soybean under CN infection [68], and in peanut exposed to nitrogen and potassium starvation [28], supporting its role in the intersection of nutrient signaling and stress responses [69]. Moreover, Pi accumulation has been shown to enhance *A. thaliana* defense against fungal pathogens by modulating the salicylic and jasmonic acid dependent defense pathways [70]. In addition, the miR399 family has been linked to broader abiotic stress responses and hormone signaling crosstalk, reinforcing its potential in enhancing plant resilience [29]. These findings suggest a novel mechanism by which nutrient signaling may contribute to nematode resistance, potentially by altering the metabolic environment of the root to deter nematode establishment or development.

Similarly, isoforms of miR408 and miR398 were upregulated in the early stages of *M. arenaria* infection in *A. stenosperma* roots. This mirrors findings in *A. thaliana* and *S. lycopersicum*, where the SPL7/miR408/miR398 regulatory module modulates both the development of nematode-induced feeding sites and copper deficiency responses [71]. Beyond nematode interactions, both miR408 and miR398 families have been associated with responses to various biotic and abiotic stresses, including fungal pathogens [72], mechanical wounding, and insect herbivory [73], highlighting their central roles in orchestrating adaptive stress responses across multiple environmental challenges.

While miR408 was upregulated, one of its targets, laccase 3, was downregulated in *A. stenosperma* during the RKN infection. Under stress conditions, miR408 induction can lead to laccase downregulation, thereby reallocating copper to other essential cuproproteins and ensuring sufficient copper for vital processes, such as energy production and defense-related processes, including oxidative stress mitigation and hormone signaling [50]. Similar mechanisms have been described in *Arabidopsis* under copper deficiency, where miR408-mediated suppression of laccases prioritizes copper for plastocyanin and other vital proteins [74].

The cytochrome P450, a non-canonical target of the miR166 family, is slightly upregulated in the resistant *A. stenosperma*, while the opposite expression pattern was observed in the more susceptible *A. duranensis*. Cytochrome P450 enzymes are crucial components of plant biochemical defense pathways and have been implicated in nematode resistance, as shown in expression and functional studies in tomato and soybean challenged with *Meloidogyne* spp. [75,76]. These enzymes participate primarily in the phenylpropanoid biosynthetic pathway, contributing to the production of lignin and other phenolic compounds that fortify the cell wall and restrict nematode invasion [77].

Additionally, differentially expressed members of the miR166 and miR396 families were found to target genes encoding pentatricopeptide repeat (PPR) proteins downregulated in RKN-infected *A. stenosperma* roots. PPR proteins are known to regulate RNA processing and play significant roles in plant responses to both biotic and abiotic stresses [44]. Studies have shown that knockout of PPR genes can lead to increased reactive oxygen species (ROS) accumulation, lipid peroxidation, and enhanced superoxide dismutase activity, collectively contributing to improved defense responses and stress adaptation [78,79]. These findings suggest that miRNA-mediated regulation of cytochrome P450s and PPR proteins may be integral to the enhanced resistance observed in *A. stenosperma*.

Most interestingly, three miRNAs predicted to target NLR-type resistance (R) genes in *A. stenosperma* (miR482a, miRNOVO12 and miRNOVO 13) were downregulated during *M. arenaria* infection while their corresponding NLR genes were upregulated. NLRs are central components of plant innate immunity, functioning as intracellular immune receptors, detecting pathogen-associated molecular patterns (PAMPs) or specific effector molecules and initiating robust downstream defense signaling [80]. Several studies have demonstrated that miRNAs contribute to the regulation of immune homeostasis by targeting NLRs and related resistance genes highlighting their role in balancing growth and defense in plants [81,82,83].

Among the *A. stenosperma* miRNA/R-genes modules identified, miR482/CCNL module is particularly noteworthy, as miR482 family is known to target conserved regions within NLR resistance genes, initiating the production of phased secondary small interfering RNAs (phasiRNAs), which serve to amplify the silencing signal [81]. The miR482-mediated silencing cascade has shown to be suppressed in plants infected with fungi, viruses, bacteria and RKNs [27,84,85], allowing pathogen-inducible expression of NBS-LRR proteins, and therefore contributing to a new layer of defense against pathogen attack. This miRNA-mediated regulation of NLR genes has been described in several plant families, including Solanaceae and Fabaceae, supporting the notion that this is a conserved mechanism of immune modulation across angiosperms [86].

Additionally, both miRNOVO12 and miRNOVO13 were downregulated in *A. stenosperma* during the early stages of infection (3 and 6 DAI), while their respective targets, TNL and TNX genes, were upregulated. In contrast, these targets were suppressed in the susceptible *A. duranensis*. These findings suggest that these novel miRNAs identified in *A. stenosperma* may act as a regulator of NLR-mediated defense responses in the resistant wild species and underscore the diversification of miRNA regulatory networks contributing to nematode resistance in *Arachis*.

In this study, the comparative analysis of miRNA and mRNA expression profiles between contrasting genotypes for RKN resistance revealed several miRNA–target regulatory pairs that may contribute to the distinct resistance phenotypes observed in the two species. However, despite the significant advances in bioinformatics, accurately predicting genuine miRNAs and their targets remains a major challenge due to the complexity of miRNA–mRNA interactions and context-dependent regulation. To complement computational predictions, small RNA–based approaches such as artificial microRNAs (amiRNAs) have been developed for functional validation and for studying the regulatory control of gene expression associated with plant stress responses [87]. These tools are particularly valuable for fine-tuning the expression of NLR genes, whose excessive accumulation can lead to autoimmunity, or cell death, emphasizing the need for precise regulation to maintain plant fitness [88]. In this study, twelve miRNAs, including four novel ones, were identified as potential regulators of NLRs in wild *A. stenosperma*. These miRNA–mRNA pairs represent promising candidates for in planta validation and for the development of RNA-based biotechnological strategies to enhance disease resistance in peanut and other legume crops.

## 4. Materials and Methods

### 4.1. Plant Materials and Nematode Bioassays

Seeds of the wild RKN-resistant *A. stenosperma* (accession V10309) were obtained from the Active Germplasm Bank of Embrapa Genetic Resources and Biotechnology (Cenargen, Brasília, Brazil). For the *A. stenosperma* miRNA libraries, a randomized complete block design of 30 plants (3 blocks of 10) was produced and grown under greenhouse conditions. When four-weeks-old, half of the plants in each block were inoculated with 2500 juveniles (J2) of *M. arenaria* and the other half with deionised water according to Morgante et al., [89]. Roots from inoculated and mock control were collected six days after inoculation (6DAI). Root samples were immediately frozen in liquid nitrogen and kept at −80 °C for miRNA and total RNA extractions.

### 4.2. miRNA Extraction and Sequencing

Three biological replicates were prepared for each treatment, consisting of pooled root samples from three individual *A. stenosperma* plants. These included roots inoculated with *M. arenaria* (AsINOC) and their corresponding non-stressed controls (AsCTR). Each pooled sample was divided into two groups: one designated for miRNA extraction and the other for total RNA extraction, ensuring parallel analyses of small RNA and transcriptome profiles from the same biological material.

Plant miRNA was extracted using mirVana™ miRNA Isolation Kit (ThermoFischer Scientific, Waltham, MA, USA, Cat. No. AM1560) [90] and used to produce six miRNA libraries (3 AsINOC, 3 AsCTR) using the NEBNext Small RNA Sample Prep SET for Illumina kit (New England BioLabs, Hitchin, UK, Cat. No.50-202-4020). All six libraries and their technical replicates were constructed and single-end sequenced on a HiSeq4000 System using the service of the University of Illinois (EUA). Transcriptomic raw data is available on NCBI Bioproject PRJNA284674 under accession numbers SAMN49651445 to SAMN49651450.

### 4.3. miRNA Identification and Target Prediction

As there is not yet a fully annotated genome for *A. stenosperma* available, high-quality, adapter-trimmed reads were aligned to the *A. duranensis* reference genome (version 1.0; https://peanutbase.org/, accessed on 29 October 2025) using Kallisto v0.46.1 with default parameters [91]. Adapter sequences were removed and reads between 20 and 24 nucleotides in length were selected using Cutadapt v4.6 [92]. To eliminate non-miRNA contaminants, such as rRNAs, tRNAs, snRNAs, and snoRNAs, trimmed reads were mapped against reference sequences using Bowtie v1.2.1 [93].

*A. stenosperma* miRNA loci were then identified using both Mireap (https://github.com/liqb/mireap, accessed on 29 October 2025) and ShortStack v4.1.2 [94], with quantification and classification performed using the latter tool. Homology-based annotation of predicted miRNAs was carried out using BLASTn v2.15.0 (e-value < 1 × 10^−4^) against the miRBase database (https://www.mirbase.org/, accessed on 29 October 2025; Release 22.1) [34]. To assess the secondary structures of precursor miRNAs, folding analyzes were performed using StrucVis (https://github.com/MikeAxtell/strucVis, accessed on 29 October 2025) and the RNAfold WebServer [95].

Prediction of miRNA target genes was performed using the psRNATarget web server (http://plantgrn.noble.org/psRNATarget/, accessed on 29 October 2025) [96] with default parameters, using the *A. duranensis* genome assembly (Aradu1.1; https://peanutbase.org/, accessed on 29 October 2025) as the reference.

### 4.4. Differentially Expressed miRNAs (DEMs)

The counts for mature miRNAs from each of the three biological replicates were used for differential expression analysis using edgeR v3.40.2 [97] package. The reproducibility of the biological replicates of *A. stenosperma* samples was assessed using normalized count data obtained with the EdgeR package. These data were processed using the DESeq2 package v1.40.2, which generated a distance matrix among the six samples. Data visualization was performed using ClustVis (https://biit.cs.ut.ee/clustvis/, accessed on 29 October 2025) [98].

For the *A. stenosperma* libraries data (3 AsINOC and 3 AsCTR), miRNAs were considered differentially expressed (DEMs) if they exhibited an adjusted *p*-value < 0.05 (Benjamini–Hochberg correction) and a false discovery rate (FDR) below 5%.

The expression profiles of miRNA target genes in *A. stenosperma* were visualized using heatmaps generated with the ggplot2 package v3.5.1 [99], based on transcriptome datasets previously published by our group [12,40] from root samples of *A. stenosperma* plants infected with *M. arenaria*.

### 4.5. Expression Analysis by qRT-PCR

To validate the in silico expression analysis of miRNAs and their putative target genes, quantitative RT-PCR (qRT-PCR) was performed using the same three biological replicates used for miRNA sequencing. Equal amounts of total RNA per sample were pooled from three plants to produce three independent biological replicates.

For miRNA expression analysis, the stem-loop quantitative RT-PCR (qRT-PCR) was conducted essentially as in Varkonyi-Gasic et al. [36]. Stem-loop reverse transcription specific primers were designed based on the miRNA sequences identified here, and Universal primers were designed as downstream primers based on the stem-loop structure (Supplementary Table S4). Reverse transcription reactions were performed using 2 µg of total RNA and M-MLV Reverse Transcriptase kit (Promega, Madison, WI, USA, Cat. No. M1701) at 16 °C for 30 min, followed by 60 cycles at 30 °C for 30 s, 42 °C for 30 s, 50 °C for 1 s and terminated by incubating at 85 °C for 5 min, according to Chen et al. [100].

For target genes expression analysis, total RNA was extracted from root samples using the Quick-RNA Plant Miniprep Kit (Zymo Research, Irvine, CA, USA, Cat. No. R2024), according to the manufacturer’s instructions. Next, DNA contamination was eliminated and cDNA synthesized as previously described [90].

For both miRNAs and mRNA samples, qRT-PCR reactions were performed with three biological replicates and two technical replicates on a StepOne Plus Real-Time PCR System (Applied Biosystems, Foster City, CA, USA) using specific primers (Supplementary Table S3) as previously described [89]. The online real-time PCR Miner tool [101] was used to estimate primer efficiency and optimal average cycle of quantification (Cq) values. The relative quantification (RQ) of miRNAs and target gene mRNA levels was determined and statistically tested using the REST 2009 v. 2.0.13 software [102]. The RQs of miRNA levels were normalized with the small nuclear RNA (snRNA) U3 and U6 [100,103], and for target genes with the 60S and GAPDH reference genes (Supplementary Table S3), in accordance with [36] and [104], respectively.

## 5. Conclusions

In summary, our results reveal an intricate miRNA/target regulatory network in *A. stenosperma* contributing to enhanced resistance against *M. arenaria*. This network integrates transcription factors, NLR immune receptors, RNA-binding proteins, cell wall-associated proteins, and components of hormone and redox signaling pathways. The modulation of miRNA expression represents a promising strategy to enhance crop resilience to biotic stresses. This can be achieved through overexpression of defense-promoting miRNAs or their targets [105,106], or through the use of artificial miRNAs (amiRNAs) for targeted gene silencing [107,108]. Additionally, CRISPR/Cas9 genome editing allows precise modification of miRNA loci or their target binding sites, enabling fine-tuning miRNA-mediated regulatory networks [109]. Elucidating the miRNA-mediated regulatory mechanisms in stress-resilient wild *Arachis* species holds great promise for developing peanut cultivars with durable and broad-spectrum resistance.

## Data Availability

The data that support the reported results can be found at the NCBI Sequence Read Archive (SRA) database (NCBI Bioproject PRJNA284674 under accession numbers SAMN49651445 to SAMN49651450).

## Supplementary Materials

The following supporting information can be downloaded at: https://www.mdpi.com/article/10.3390/ijms262210824/s1.

## Abbreviations

The following abbreviations are used in this manuscript:

miRNAs	MicroRNAs
DEMs	Differentially Expressed MicroRNAs
NLR	Nucleotide-binding site Leucine-Rich
RKN	Root-Knot Nematode
mRNA	Messenger RNA
PR	Pathogenesis-related genes
R	Resistance genes
AsCTR	Control samples (not inoculated) of *Arachis stenosperma*
AsINOC	Inoculated samples of *Arachis stenosperma*
Mare	Sample of *Meloidogyne arenaria*
Mb	Million base pairs
nt	Nucleotides
miRNOVO	New miRNA candidates
qRT-PCR	Quantitative Reverse Transcription-Polymerase Chain Reaction
RNA-Seq	RNA sequencing
DAI	Days After Inoculation
MLP	Major Latex Protein
MLO	Mildew Locus O
TF	Transcription Factor
SPL	Squamosa Promoter-Binding Protein-Like
HD-ZIP	Homeodomain-leucine Zipper
FC	Fold-change
TCP2	Teosinte branched1/Cycloidea/Proliferating cell factor
PHO2	Phosphatase2
DEG	Differentially Expressed Gene
UV	Ultraviolet
TIR-NBS-LRR	Toll-Interleukin Receptor /Nucleotide-Binding Site/Leucine-Rich Repeat
PPR	Pentatricopeptide Repeat
CN	Cyst Nematode
GRF	Growth Regulating Factor
Pi	Phosphate
ROS	Reactive Oxygen Species
PAMP	Pathogen-Associated Molecular Patterns
CCNL	Cyclin L
phasiRNA	Phased secondary small interfering RNAs
TNL	TIR-NBS-LRR
MYB	Myeloblastosis
TNX	Toll-Interleukin Receptor /Nucleotide-Binding Site without LRR domain
AP2	APETALA
amiRNA	Artificial miRNA
CRISPR	Clustered Regularly Interspaced Short Palindromic Repeats
cv	Cultivate
